# Modulation of GABAergic System in a Chicken Retinal Ischemic Model: The Role of Chloride Cotransporters

**DOI:** 10.1002/jnr.70043

**Published:** 2025-05-12

**Authors:** A. A. Nascimento, V. S. Miya‐Coreixas, D. S. M. Araújo, T. H. O. Nascimento, G. F. Santos, R. Brito, K. C. Calaza

**Affiliations:** ^1^ Laboratory Neurobiology of the Retina, Department of Neurobiology and Program of Neurosciences, Biology Institute Fluminense Federal University Rio de Janeiro Brazil; ^2^ Laboratory Neurobiology of the Retina, Department of Neurobiology and Program of Biomedical Sciences, Biology Institute Fluminense Federal University Rio de Janeiro Brazil; ^3^ Laboratory of Neural Physiology and Pathology, Department of Cellular and Molecular Biology, Biology Institute, Program of Neurosciences Fluminense Federal University Rio de Janeiro Brazil

**Keywords:** cell death, chloride cotransporters, GABA, oxygen–glucose deprivation

## Abstract

Retinal ischemia is a significant pathological condition that contributes to visual impairment and neuronal cell death in various retinopathies. Evidence suggests that GABA release during ischemic events may exhibit neuroprotective properties, but conflicting findings highlight a potential shift in its effects due to altered chloride ion homeostasis. This study aimed to investigate the role of the GABAergic system in retinal ischemia, focusing on the temporal dynamics of GABA release and its impact on retinal damage. We hypothesized that ischemia‐induced changes in GABA transport and chloride ion equilibrium contribute to neuronal damage, which can be mitigated by modulating GABAergic activity. Using an ex vivo chick retina model subjected to oxygen and glucose deprivation (OGD), during different times, we assessed morphological changes, cell death, GABA levels, transporter activity, and the levels of chloride cotransporters NKCC1 and KCC2. Pharmacological interventions, including picrotoxin and bumetanide, were used to evaluate neuroprotective effects. Our results revealed that OGD‐induced significant morphological changes and cell death in the retina. GABA levels were reduced in a GAT‐1‐dependent manner, while picrotoxin and bumetanide demonstrated neuroprotective effects by mitigating retinal swelling and modulating the GABAergic system. Notably, OGD increased NKCC1 content, but not KCC2 levels, indicating a disruption in chloride homeostasis. These findings suggest that ischemia‐induced alterations in GABAergic activity and chloride transport contribute to retinal damage. Targeting these pathways with pharmacological agents, such as bumetanide, may offer therapeutic strategies for mitigating ischemic retinal injury. Further research is recommended to explore the clinical applicability of these findings in the ischemic retina.

## Graphical abstract

Oxygen–glucose deprivation (OGD) promotes GABA release and increases NKCC1 levels, which change the electrochemical chloride gradient. In these circumstances, activation of ionotropic GABA receptors would lead to cell depolarization. Thus, in the early stages of ischemia, GABA intensifies glutamate excitotoxicity by an alteration in chloride transporters, worsening cell death.

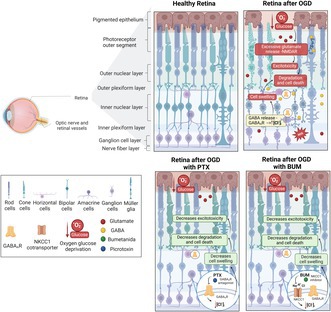

**Summary**

- Ischemia is a pervasive condition present in several retinopathies, such as diabetic retinopathy and glaucoma.
- Understanding its molecular mechanisms is a strategy to develop new protective interventions.
- In the present study, the data indicate that ischemia alters chloride cotransporters, possibly turning GABA into a depolarizing signal likely to increase excitability, promoting cell death by excitotoxicity.
- So, although GABA is the main inhibitory neurotransmitter in the retina, after acute ischemia it becomes harmful.
- Thus, modulating GABA can be a strategy for neuroprotection, but the temporal course of the treatment must be carefully designed.

## 1 Introduction

Retinal ischemia is a prevalent pathological condition and a key contributor to visual impairment. It is a central mechanism driving neuronal cell death in various retinal diseases, including glaucoma, diabetic retinopathy, age‐related macular degeneration, and retinopathy of prematurity. Despite extensive research on ischemic damage, the role of specific neurotransmitter systems, particularly the GABAergic system, in retinal ischemia remains insufficiently explored (Pereira‐Figueiredo et al. 2022).

While the function of the GABAergic system in the brain is well‐documented, its role in the ischemic retina is less understood. GABA, predominantly localized in the amacrine cells of the inner nuclear layer (INL), displaced amacrine cells in the ganglion cell layer, horizontal cells, and the inner plexiform layer (IPL), is released by excitatory stimulation and during ischemic events (Osborne and Herrera 1994; Rego et al. 1996; Calaza et al. 2001, 2006). Notably, ischemia triggers significant alterations in GABA distribution, with evidence of its accumulation in Müller cells in vascular retinas, potentially mediated by GABA‐transaminase inhibition. The mechanism of GABA release during ischemia may involve transporter reversal, independent of calcium influx, suggesting a complex role in retinal homeostasis (Barnett and Osborne 1995; Kobayashi et al. 1999).

Although GABA release during ischemic insults has been proposed as neuroprotective—by counteracting excessive neuronal excitation driven by glutamate—emerging evidence points to a dual role. Changes in chloride ion (Cl^−^) equilibrium during ischemia may alter GABA's effects from inhibitory to excitatory, exacerbating cellular damage (Nascimento et al. 2024). These changes are associated with the dysregulated activity of cotransporters NKCC1 and KCC2, which maintain the Cl^−^ electrochemical gradient (Owens and Kriegstein 2002; Payne et al. 2003; Arroyo et al. 2013). Ischemic conditions in mature central nervous system, including the retina, induce transporter expression patterns resembling those of immature stages, potentially causing intracellular Cl^−^ accumulation and shifting GABA responses from hyperpolarization to depolarization (Ben‐Ari 2002; Ben‐Ari et al. 2007; Vardi and Zhang 2009; Kim and Ahn 2012; Ben‐Ari and Cherubini 2022; Hartmann and Nothwang 2022; Yan et al. 2001, 2003). This dichotomy underscores the importance of exploring the temporal and mechanistic parameters of GABA's action during ischemia.

In this study, we hypothesize that GABA‐mediated responses during ischemia are temporally and mechanistically dependent on changes in transporter expression and Cl^−^ equilibrium. To address this, we employed an ex vivo model of retinal ischemia using oxygen and glucose deprivation (OGD) to assess the morphological and molecular changes associated with GABA signaling. Specifically, we investigated how OGD influences retinal GABA content, GAT‐1‐mediated transport, and the expression of Cl^−^ cotransporters. Additionally, we evaluated the neuroprotective effects of pharmacological agents such as picrotoxin (PTX) and bumetanide in mitigating ischemia‐induced damage.

This approach aims to elucidate the multifaceted role of GABA in ischemic retinopathy and to identify potential therapeutic strategies for preserving retinal function under pathological conditions.

## 2 Materials and Methods

### 2.1 Reagents

Entellan new (cat# 1.07961), NNC‐711 hydrochloride (cat# N142), bicuculine (cat# 14340), picrotoxin (cat# P1675), bumetanide (cat# B3023), bovine serum albumin (BSA, cat# A2153), 3,3‐diaminobenzidine (DAB, cat# D5637), antitubulin (cat# T5168, RRID:AB_477579), and anti‐GABA (cat# A2052, RRID:AB_477652) antibodies were purchased from Sigma‐Aldrich (St. Louis, Missouri, USA). Anti‐rabbit (cat# BA‐1000, RRID:AB_2313606) and anti‐goat (cat# BA‐5000, RRID:AB_2336126) biotinylated antibodies, and Vectastain Elite ABC‐HRP kit were obtained from Vector Laboratories (Burlingame, CA, USA). Antibodies anti‐GAT‐1 (cat# AB1570W, RRID:AB_90791) and anti‐KCC2 (cat# 07‐432, RRID:AB_310611) were purchased from Chemicon/Millipore (Billerica, MA, USA). PVDF membrane (cat# 10600023), ECL prime kit (cat# RPN2232) and HRP‐conjugated secondary antibodies anti‐rabbit (cat# NA934, RRID:AB_772206) and anti‐mouse (cat# NA931, RRID:AB_772210) were obtained from Amersham/Cytiva (Little Chalfont, United Kingdom). Dimethyl sulfoxide (DMSO, cat# D12345), bicinchoninic acid (BCA) assay (cat# 23227), and HRP‐conjugate anti‐sheep secondary antibody (cat# 61‐8620, RRID:AB_2533942) were obtained from Invitrogen/Thermo Fisher Scientific (Waltham, MA, USA). CytoTox 96 Non‐Radioactive Cytotoxicity Assay colorimetric kit was purchased from Promega (Madison, WI, USA). cOmplete Mini cocktail protease inhibitor was obtained from Roche (Germany). Antibody anti‐NKCC1 (cat# T4, RRID:AB_528406) was obtained from Developmental Studies Hybridoma Bank (DSHB, Iowa City, IA, USA). Antibody anti‐GAD 65/67 was produced and kindly provided by Dr. Oertel.

### 2.2 Animals

All experimental procedures conducted at the Federal Fluminense University complied with the guidelines established by the Animal Research Ethics Committee (CEUA/Proppi/UFF) under project approval number 197/2012. Fertilized eggs from the White Leghorn breed (*Gallus domesticus*) were sourced from a local farm in Rio de Janeiro, RJ, Brazil. The eggs were incubated in a manual incubator (Chocadeira Manual, Premium Ecológica, Belo Horizonte, MG, Brazil) at a stable temperature of 37.8°C, with humidity levels maintained at 30°C as measured by a wet thermometer, until hatching. Newly hatched chicks, aged between 0 and 7 days (P0–P7), were housed in appropriate cages with free access to food and water until the experimental procedures began. The experiments were primarily performed in the afternoon, and the time of euthanasia was at the end of the afternoon. All animals were maintained on daily cycles of 12 h of light and darkness with access to food and water ad libitum. Animals aged postnatal days 2 to 7 (P2–P7) were randomly selected for experiments involving ex vivo retinal preparations subjected to OGD, whereas each experiment used animals at the same age. Although the retinas of the animals in this age range are already considered mature, sex differentiation was not performed due to the animals' young age and lack of visible sexual dimorphism at this stage of development.

### 2.3 OGD in Retinas Ex Vivo

Posthatched chicks were euthanized by decapitation, and their eyes were carefully removed to isolate the retinas, which were then sectioned along the sagittal plane. The posterior segment of the eye, including the retinal pigment epithelium (RPE), was utilized for the OGD procedure. The eye cups from both eyes were divided into two, three or four segments of comparable size that were randomly assigned to the different experimental groups.

For cell viability assay, by LDH activity measure, and protein analysis, via western blotting, retinas were separated from the RPE by dissection in calcium‐ and magnesium‐free medium (CMF) at 37°C. In the control condition, the retinal segment was incubated in a Ringer's solution (120 mM NaCl, 3 mM KCl, 1 mM NaH_2_PO_4_, 30 mM NaHCO_3_, 1 mM CaCl_2_, 1 mM MgCl_2_.6H_2_O, and 10 mM glucose). In contrast, OGD conditions were achieved by incubating retinal segments in a Ringer solution with no glucose. The control and OGD Ringer were aerated for 15 min before the experiment began, respectively with a gaseous mixture of 95% O_2_ and 5% CO_2_ or 95% N_2_ and 5% CO_2_. The pH of both solutions was adjusted to 7.2–7.4.

Retinal segments were subsequently incubated in either control or OGD solution for 15, 30, or 50 min. During the incubation, the solutions were continuously supplied with the appropriate gaseous mixture (control: 95% O_2_ and 5% CO_2_ and OGD: 95% N_2_ and 5% CO_2_) and maintained at 37°C in a water bath. For experiments evaluating the effect of NNC‐711, retinal segments were preincubated with the drug under control conditions for 10 min before transitioning to the OGD condition, with the drug remaining present throughout. The other experimental groups of this experiment were preincubated in the same conditions but with no NNC‐711.

All drugs used in the experiments were stored at −20°C. In experimental conditions where drugs were absent, the same proportion of the vehicle used for drug preparation was added to ensure consistency across conditions. Bicuculline was dissolved in DMSO, while Picrotoxin and bumetanide were dissolved in ethanol.

### 2.4 Nissl Staining and Immunohistochemistry

Retinal segments were fixed by immersion in 4% paraformaldehyde prepared in 0.16 M phosphate buffer (pH 7.2) for 2 h, followed by rinsing with phosphate buffer 0.16 M. To ensure cryoprotection, the retinas were subjected to a sucrose gradient (10%, 20% and 30%) and subsequently embedded in optimal cutting temperature compound (OCT) (Sakura Finetek, Torrance, CA, USA). The samples were then frozen on dry ice. Transverse retinal sections (10 μm thick) were collected on poly L‐lysine‐coated slides and stored at −20°C. On the day of histological processing, sections were thawed and equilibrated to room temperature. To minimize technical variability and ensure the reliability of the comparative analysis, both control and treated retinal samples of the same experiment were collected on the same slide. This strategy ensured that observed differences could be attributed to the specific treatments. For Nissl staining, the sections were washed with distilled water, stained with 0.25% cresyl violet, and subjected to an ethanol gradient, followed by two immersions in xylol. Finally, the slides were mounted using Entellan mounting medium.

For immunohistochemistry, the sections were washed three times with phosphate‐buffered saline (PBS: KCl 2.7 mM, KH2PO4 1.47 mM, NaCl 136 mM, Na2HPO4.7H2O 8 mM; pH 7.4) and incubated for 1 h in 5% bovine serum albumin (BSA) to block nonspecific binding. The sections were then incubated overnight with either polyclonal anti‐GABA (1:7000) or polyclonal anti‐GAT‐1 (1:100). On the following day, the sections were washed with PBS and incubated for 2 h with secondary antibodies: antirabbit (1:200, BA‐1000) or antigoat (1:200). Afterward, the sections were washed and incubated with 1:50 avidin–biotin complex (ABC) for 1.5 h. After additional washes with PBS, the sections were incubated with 0.05% 3,3‐diaminobenzidine (DAB) and 0.01% hydrogen peroxide for 10 min to visualize the antigen–antibody complex. Finally, the sections were mounted using a solution of 40% glycerol in 0.2 M phosphate buffer, diluted in PBS containing 0.25% Triton X‐100.

### 2.5 Analysis of Retinal Sections

Retinal morphology and thickness in the central and peripheral regions were analyzed using digital photomicrographs acquired with a DFC310 FX or DFC350 FX digital cameras (Leica Microsystems, Wetzlar, Germany), through differential interference contrast (DIC) microscopy (DM2500, 40× objective, 10× ocular, Leica Microsystems, Wetzlar, Germany). A minimum of five microscopic fields were examined along the entire extent of each retinal section, analyzing at least three distinct retinal sections (≥ 40 μm apart). Nissl‐stained sections were employed to qualitatively evaluate retinal morphology, focusing on the presence of vacuoles, edematous cell profiles, and alterations in the thickness and organization of retinal layers.

Digital photomicrographs were captured using an SV Micro camera (Sound Vision, USA) under light microscopy (Axioskop, 40× objective, 10× ocular, Zeiss, Germany). The thickness of the inner plexiform layer (IPL) was measured as the distance between its innermost and outermost boundaries using the “line” tool in the Image J software, with a system spatial resolution of 7.0 pixels/μm. Immunostaining intensities for GABA and GAT‐1 were analyzed following a previously established protocol by the research group (Calaza et al. 2001; Guimarães‐Souza et al. 2011). Digital images of retinal slices immunostained for GABA or GAT‐1 were converted to grayscale (8‐bit), and pixel intensity values were detected within a defined tonal scale (0–255). The IPL was divided into five equal segments, and the optical density of each segment (S1–S5) was measured. Background staining intensity within the same field was also assessed, and the staining intensity from the region expected to have the respective immunolabeling, GABA or GAT‐1, was subtracted from the intensity of a region that does not express GABA or GAT‐1, such as the outer third of the inner nuclear layer (INL). The number of GABA‐immunolabeled (GABA‐positive) cell bodies in the INL and/or ganglion cell layer (GCL) was quantified using a handheld counter under DIC‐conjugated light or fluorescence microscopy. To specifically quantify GABA‐positive amacrine cells in the INL, immunolabeled cells in the three innermost rows of the INL were counted.

### 2.6 Western Blotting

Isolated retinal segments were collected in RIPA buffer (NaCl 150 mM, Tris‐base 50 mM, EGTA 5 mM, Triton X‐100 1%, DOC 0.5%, SDS 0.1%, pH 7.5) containing protease inhibitors and dithiothreitol 1 mM (DTT). The samples were homogenized, and protein levels were determined using the bicinchoninic acid (BCA) assay. The resulting supernatants were collected and stored in a denaturing buffer (Tris–HCl 0.5 M/0.4% SDS pH 6.8, glycerol 30%, SDS 10%, DTT 0.6 M, bromophenol blue 0.01%). Samples were heated to 100°C for 5 min and stored at −20°C. The denatured proteins (30 μg) were subjected to electrophoresis on a polyacrylamide‐SDS gel (4%–10%) and subsequently transferred to a PVDF membrane. The block solution was 5% skimmed milk diluted in Tris +0.1% Tween. The membranes were then incubated overnight at 4°C with primary antibodies anti‐NKCC1 (1:1000, T4), anti‐KCC2 (1:500), anti‐GAD 65/67 (1:500), and anti‐α‐tubulin (1:70,000) with continuous agitation. Following primary antibody incubation, membranes were washed three times, incubated with secondary antibodies, and washed with TBS‐T. Protein detection was performed using the ECL Prime kit, and images were captured using the ChemiDoc MP System (BioRad, Hercules, CA, USA, RRID:SCR019037). The ImageJ program was used for quantification (Version 1.53, NIH, USA, RRID:SCR 003070). To normalize protein expression levels, α‐tubulin was used as a loading control. Although α‐tubulin expression can be affected by ischemic conditions, we carefully assessed tubulin levels under OGD conditions. No significant changes in tubulin expression were observed across the experimental groups. Additional experiments were performed to compare α‐tubulin levels at different time points and experimental conditions, ensuring that observed differences in protein content were not due to variations in α‐tubulin levels.

### 2.7 Cell Viability Assay

Cell viability was assessed using the CytoTox 96 Non‐Radioactive Cytotoxicity Assay colorimetric kit, which measures lactate dehydrogenase (LDH) activity. Retinal segments were collected in lysis solution at room temperature for 30 min and then frozen at −20°C, corresponding to the intracellular fraction. The respective solutions were collected, frozen, and corresponded to the extracellular fraction. After thawing, the samples were homogenized and centrifuged at 2000 rpm (approximately 35.8 G) for 4 min at 25°C. LDH activity was quantified from the intracellular and the extracellular fraction, with duplicate measurements for each. Colorimetric detection was performed at 490 nm using a microplate reader (iMARKTM, BioRad, Hercules, CA, USA, RRID:SCR023799). The average absorbance values from each condition were used to determine the LDH activity in both fractions. Total LDH activity was calculated by summing the values from the intracellular and extracellular fractions. The percentage of LDH released was calculated by dividing the extracellular fraction value by the total LDH activity, with adjustments made for any necessary dilution factors.

### 2.8 Statistical Analysis

The results are presented as the mean ± standard deviation (SD) and are expressed as a percentage of the control, IPL thickness in micrometers (μm), or optical density in arbitrary units (a.u.). Statistical analysis was performed using Student's *t*‐test, one‐way ANOVA with Bonferroni posttest, or two‐way ANOVA with Bonferroni posttest, utilizing GraphPad Prism (version 8.0, RRID:SCR002798). Statistical significance was set at *p* < 0.05.

## 3 Results

### 3.1 OGD Induces Morphological Changes and Cell Death in Chick Retina Ex Vivo

**FIGURE 1:**
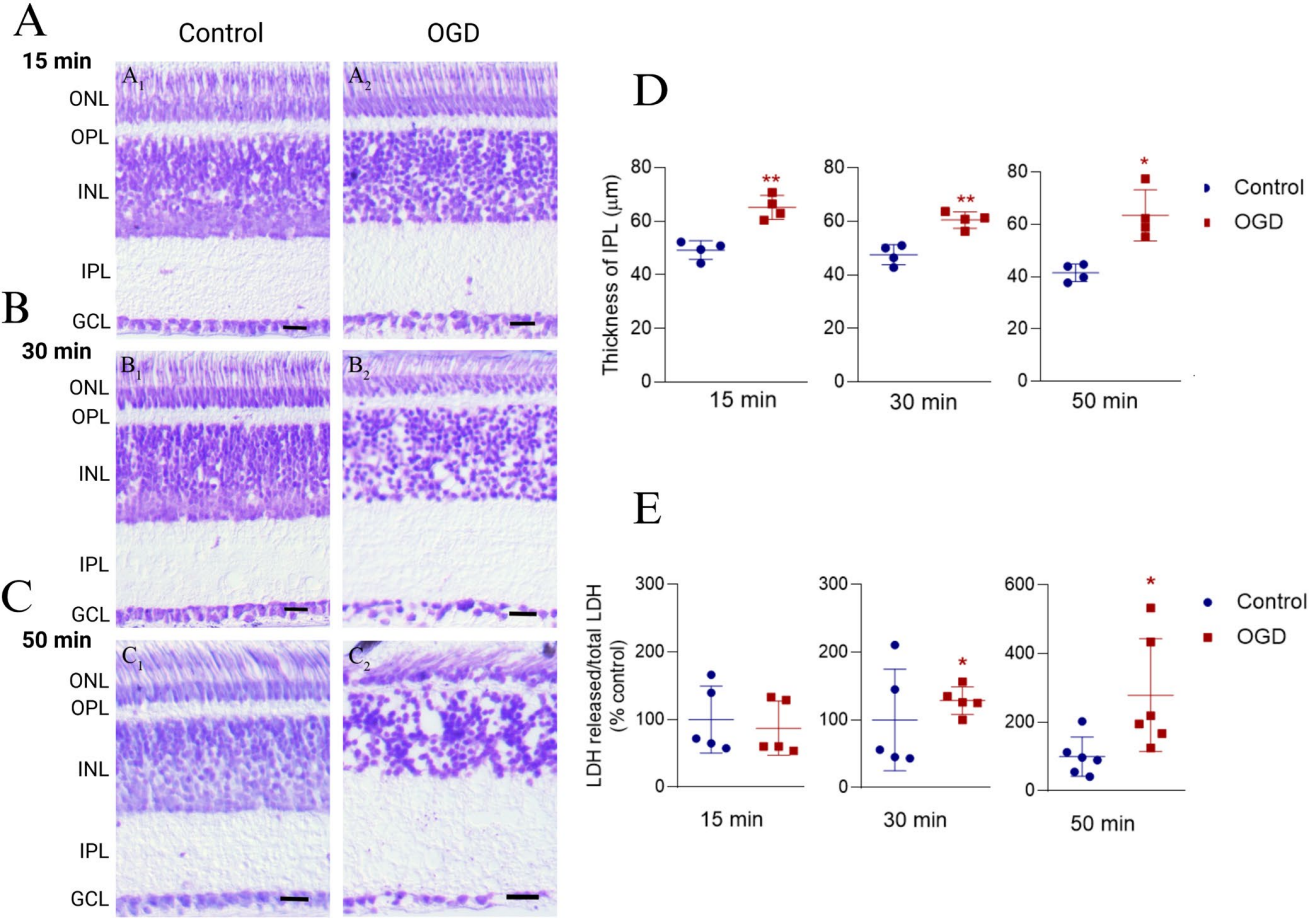
OGD induces morphological changes and cell death in chick retina ex vivo. (A–C) Photomicrographs of retinal sections subjected to Nissl staining. Retinal segments were exposed to OGD for 15 (A1‐2), 30 (B1‐2), or 50 min (C1‐2). Qualitative analysis indicates that OGD, in a time‐dependent manner, led to progressive disorganization of the nuclear layers and vacuolation of the ONL and INL, indicative of cell death and swelling in the IPL. (D) IPL thickness (μm) was measured as a morphological parameter of retinal swelling. The graph shows a significant increase in IPL thickness in retinal segments subjected to OGD at all analyzed time points. Values are expressed as mean ± standard deviation (SD). Control: 15 min (49.26 ± 3.45 μm, *n* = 4), 30 min (47.52 ± 3.76 μm, *n* = 4), 50 min (41.52 ± 3.36 μm, *n* = 4). OGD: 15 min (65.16 ± 4.48 μm, *n* = 4), 30 min (60.49 ± 3.00 μm, *n* = 4), 50 min (63.46 ± 9.77 μm, *n* = 4). Statistical analysis: 15 min, *t*(6) = 4.78, *p* = 0.003; 30 min, *t*(6) = 4.41, *p* = 0.004; 50 min, *t*(6) = 3.53, *p* = 0.012. (E) Cell death was assessed by measuring LDH activity in the extracellular medium, expressed as the ratio of released LDH to total LDH (% of control) in retinas subjected to OGD (oxygen–glucose deprivation). The graph shows a significantly higher percentage of LDH release from retinas exposed to OGD for 30 or 50 min. Values are presented as mean ± standard deviation (SD). Control: 15 min (100 ± 49.44%, *n* = 5), 30 min (100 ± 75.00%, *n* = 5), 50 min (100 ± 56.89%, *n* = 6). OGD: 15 min (87.18 ± 40.21%, *n* = 5), 30 min (128.50 ± 20.32%, *n* = 5), 50 min (278.90 ± 164.60%, *n* = 6). Statistical analysis: 15 min, *t*(8) = 0.89, *p* = 0.401; 30 min, *t*(8) = 3.92, *p* = 0.004; 50 min, *t*(10) = 3.85, *p* = 0.003. GCL, ganglion cell layer; INL, inner nuclear layer; IPL, inner plexiform layer; LDH, lactate dehydrogenase; ONL, outer nuclear layer; OPL, outer plexiform layer. Significance: *p < 0.05 and **p < 0.01 compared with control. Scale bar = 20 μm.

The first goal of this study was to characterize the morphological changes induced by OGD in the mature chicken retina ex vivo. Nissl staining of retinal cross‐sections was performed to examine morphological alterations in retinas subjected to OGD for different durations (Figure 1). Significant changes were observed in the retinas exposed to OGD for 30 min (Figure 1B2) or 50 min (Figure 1C2) compared to control retinas maintained under normal conditions (Figure 1B1,C1). These changes included disorganization of the outer nuclear layer (ONL) and inner nuclear layer (INL), the presence of vacuoles in these layers, and an increase in the thickness of the inner plexiform layer (IPL). Retinas exposed to OGD for 15 min showed an increase in IPL thickness (Figure 1D) but did not exhibit notable disorganization of the nuclear layers or loss of cell bodies (Figure 1A). In addition to qualitative analysis, IPL thickness (μm) was quantitatively measured using digital images obtained from cross‐sections of retinal segments exposed to different experimental conditions. The analysis revealed an approximately 20 μm increase in IPL thickness across all time points studied (Figure 1D). Interestingly, the extent of IPL thickening was consistent across different OGD durations, suggesting that OGD induces an early and pronounced ionic imbalance, with further accentuation of this morphological change not being prominent at longer durations. To assess cell death, we measured lactate dehydrogenase (LDH) in the extracellular medium. Figure 1E shows a significant increase in LDH released from retinal segments exposed to OGD for 30 min and a robust increase after 50 min of OGD, indicative of cell death. However, no significant changes were detected during the first 15 min of OGD.

### 3.2 Retinal GABA Content Is Modified by OGD

**FIGURE 2:**
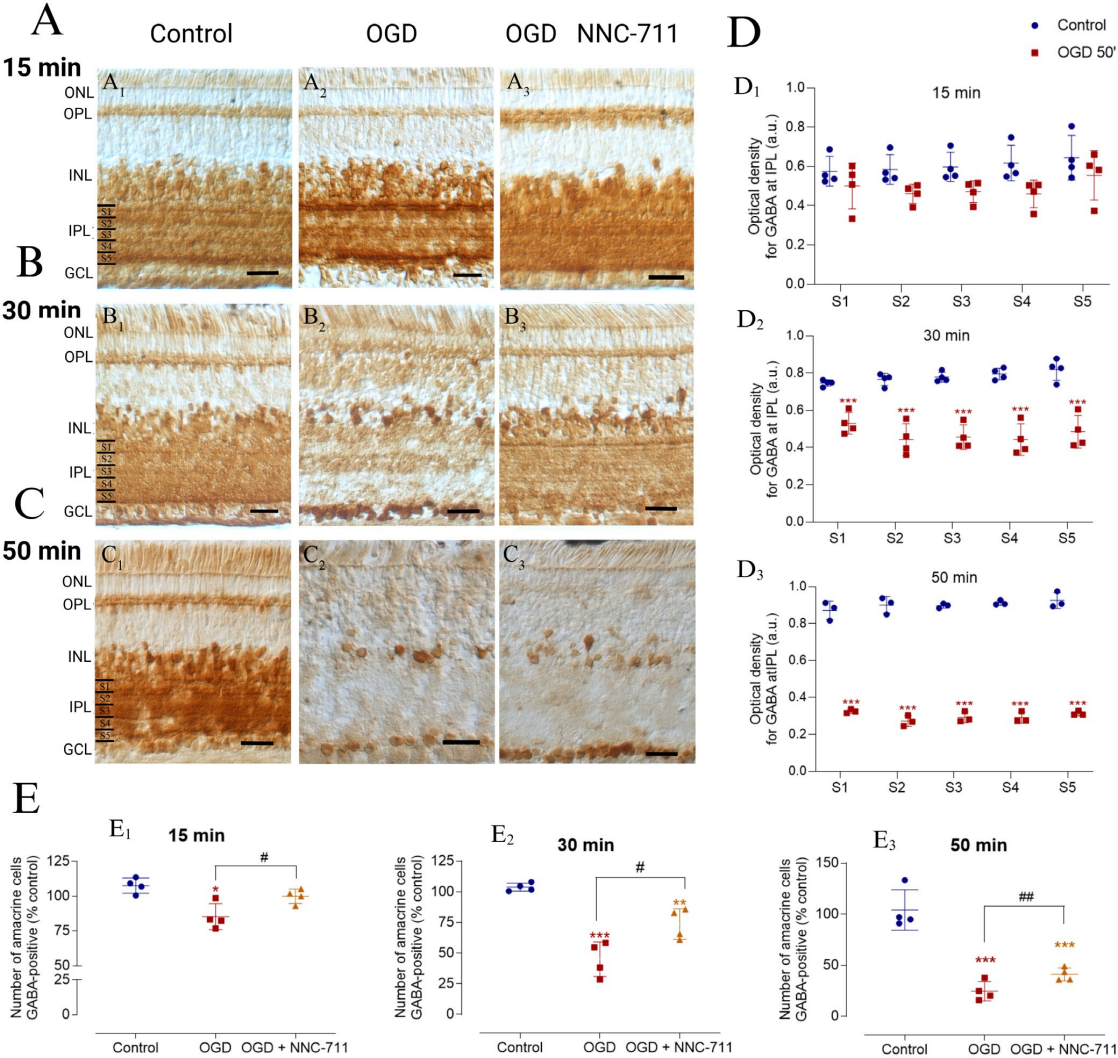
OGD reduced GABA content in the retina ex vivo in a partially GAT‐1‐dependent manner. (A–C) Photomicrographs of GABA immunohistochemically stained retinal sections. Retinal segments were subjected to OGD for 15 (A1–3), 30 (B1–3), or 50 min (C1–3) in the presence or absence of NNC‐711 (100 μM). (D1–3) Graphs show the optical density (a.u.) of GABA in each of the five sublaminae (S1–S5) of the inner plexiform layer (IPL) from retinal segments subjected to control or OGD conditions. (D1) 15 min: Control (S1: 0.57 ± 0.067; S2: 0.58 ± 0.067; S3: 0.59 ± 0.067; S4: 0.61 ± 0.089; S5: 0.64 ± 0.112); OGD (S1: 0.50 ± 0.112; S2: 0.46 ± 0.045; S3: 0.47 ± 0.045; S4: 0.46 ± 0.067; S5: 0.55 ± 0.134). 30 min: Control (S1: 0.74 ± 0.022; S2: 0.76 ± 0.022; S3: 0.77 ± 0.022; S4: 0.79 ± 0.022; S5: 0.81 ± 0.045); OGD (S1: 0.53 ± 0.045; S2: 0.44 ± 0.089; S3: 0.45 ± 0.067; S4: 0.44 ± 0.089; S5: 0.48 ± 0.089). 50 min: Control (S1: 0.87 ± 0.035; S2: 0.90 ± 0.035; S3: 0.89 ± 0.017; S4: 0.91 ± 0.017; S5: 0.92 ± 0.035); OGD (S1: 0.32 ± 0.017; S2: 0.27 ± 0.016; S3: 0.29 ± 0.017; S4: 0.29 ± 0.018; S5: 0.31 ± 0.021). Values are expressed as mean ± standard deviation (SD) (*n* = 4 for all groups). Statistical analysis was performed using two‐way ANOVA. After 15 min, two‐way ANOVA showed a significant main effect of sublamina (*F*(4, 24) = 8.528, *p* = 0.0002), but no significant effects for experimental condition (*F*(1, 6) = 3.692, *p* = 0.103) or the interaction between sublamina and condition (*F*(4, 24) = 2.456, *p* = 0.073). At 30 min, there was a significant interaction between sublamina and experimental condition (*F*(4, 24) = 11.87, *p* < 0.001), with significant main effects for both sublamina (*F*(1.577, 9.460) = 6.004, *p* = 0.025) and condition (*F*(1, 6) = 59.33, *p* = 0.0003). After 50 min, a significant interaction between sublamina and condition was observed (*F*(4, 16) = 3.755, *p* = 0.024), with a significant main effect for condition (*F*(1, 4) = 884.8, *p* < 0.001), but no significant effect for sublamina (*F*(4, 16) = 2.070, *p* = 0.133). (E1‐3) Graphs show the percentage of GABA‐positive amacrine cells in the inner nuclear layer (INL) of retinas treated or untreated with NNC‐711 (GAT‐1 inhibitor) and exposed to OGD for different time points: (E1) 15 min: Control (100.00 ± 5.48%); OGD (85.33 ± 9.34%); OGD + NNC‐711 (100.00 ± 5.18%). 30 min: Control (100.00 ± 3.30%); OGD (44.94 ± 14.06%); OGD + NNC‐711 (73.69 ± 12.36%). 50 min: Control (100.00 ± 19.96%); OGD (24.36 ± 9.46%); OGD + NNC‐711 (43.24 ± 8.50%). All groups (*n* = 4). Values are expressed as mean ± SD. The mean number of amacrine cells counted per field in the control condition was as follows: 15 min = 149.62 cells, 30 min = 140.53 cells, 50 min = 159.91 cells. Statistical analysis was performed using two‐way ANOVA followed by Bonferroni's posttest. A significant interaction between experimental condition and individual subjects was observed (*F*(6, 18) = 5.130, *p* = 0.003). The experimental condition had a significant main effect at 30 min (*F*(2, 6) = 152.1, *p* < 0.001), while individual subject variability was not significant (*F*(6, 18) = 1.105, *p* = 0.414). At 50 min, both the interaction between experimental condition and sublamina (*F*(6, 18) = 14.42, *p* < 0.001) and the main effect of the condition (*F*(2, 6) = 794.2, *p* < 0.001) were significant. OGD, oxygen–glucose deprivation; Sn, sublamina; NNC‐711, GAT‐1 inhibitor. Significance: *p < 0.05, **p < 0.01, ***p < 0.001 compared with the control; ^#^ *p* < 0.05, ^##^ *p* < 0.01 compared with OGD. Scale bar: 20 μm.

Considering the abundant population of GABAergic amacrine cells in the chick retina, we investigated their response to ischemic insult induced by OGD. Initially, the immunostaining pattern for endogenous GABA was assessed in retinas exposed to OGD for different durations (15, 30, and 50 min) (Figure 2). We observed a progressive decrease in GABA immunostaining in retinal cross‐sections subjected to OGD in a time‐dependent manner, compared with the control retinas (Figure 2A2,B2,C2 vs. Figure 2A1,B1,C1). This finding was confirmed by analyzing the GABA immunostaining intensity in the IPL, which showed that the treatment (control vs. OGD) contributed to the total variance in GABA immunostaining observed in retinas exposed to OGD for 15 min (Figure 2D1). However, the posttest did not show a significant difference between the control and OGD groups when each sublamina was compared (Table 1). Moreover, significantly reduced GABA content was observed in each of the five IPL strata 30 min after OGD (Figure 2D2 and Table 1) or 50 min (Figure 2D3 and Table 1). Additionally, quantification of GABA‐positive amacrine cells revealed a reduction in the number of GABA‐positive cells in the INL at all time points studied, but no change was observed in GCL (Figure 2E1–3,F1–3). Based on these findings, we further investigated whether the decrease in endogenous GABA levels could be attributed to the changes in GABA release induced by OGD. Previous studies from our group have shown that excitatory amino acids can induce GABA release in the retina in a GAT‐1‐dependent manner (Calaza et al. 2001; Guimarães‐Souza et al. 2011; Guimarães‐Souza and Calaza 2012). Given the role of glutamate in the context of OGD/ischemia, we explored whether the decrease in endogenous GABA content was dependent on GAT‐1 activity by using a selective blocker. Retinal segments were pretreated with the GAT‐1 inhibitor, NNC‐711 (100 μM) for 10 min before being exposed to OGD and during the intervention. The effect of OGD for 15 min on the population of GABAergic amacrine cells in the INL was completely dependent on GAT‐1 (Figure 2A3,E1). However, NNC‐711 only inhibited 20%–30% of the OGD‐induced effects after 30 min (Figure 2B3,E2) or 50 min (Figure 2C3,E3) of exposure. Interestingly, the number of GABA‐positive displaced amacrine cells did not change after 15, 30, or 50 min of OGD (15 min OGD = 96.60 ± 5.36%, *n* = 3; 30 min OGD = 115.70 ± 19.81%, *n* = 3, or 50 min OGD = 99.82 ± 16.37%, *n* = 3).

**TABLE 1:** Labeling intensity values for GABA in IPL.

Time	Lamina	Control ± SD (a.u)	OGD ± SD (a.u)	*N*
15 min	S1	0.562 ± 0.050	0.445 ± 0.222	4
	S2	0.570 ± 0,058	0.437 ± 0.092	
	S3	0.580 ± 0.068	0.446 ± 0.104	
	S4	0.594 ± 0.090	0.426 ± 0.136	
	S5	0.610 ± 0.134	0.494 ± 0.242	
30 min	S1	0.747 ± 0.004	0.556 ± 0.106***	4
	S2	0.769 ± 0.014	0.489 ± 0.148***	
	S3	0.771 ± 0.028	0.487 ± 0.124***	
	S4	0.783 ± 0.028	0.482 ± 0.156***	
	S5	0.793 ± 0.106	0.525 ± 0.160***	
50 min	S1	0.871 ± 0.050	0.325 ± 0.010***	3
	S2	0.901 ± 0.045	0.272 ± 0.029***	
	S3	0.897 ± 0.012	0.292 ± 0.029***	
	S4	0.913 ± 0.014	0.291 ± 0.029***	
	S5	0.925 ± 0.043	0.314 ± 0.014***	

Abbreviations: a.u., arbitrary unit; OGD, glucose and oxygen deprivation; SD, standard deviation; Sn, sublamina n.

*** *p* < 0.001 compared with the control.

The findings of this study suggest that the decrease in endogenous GABA levels in the retina following OGD can be partially attributed to GAT‐1‐mediated neurotransmitter release. However, part of the observed reduction at 30 and 50 min is probably due to the death of GABAergic cells and the subsequent extracellular release of GABA.

**FIGURE 3:**
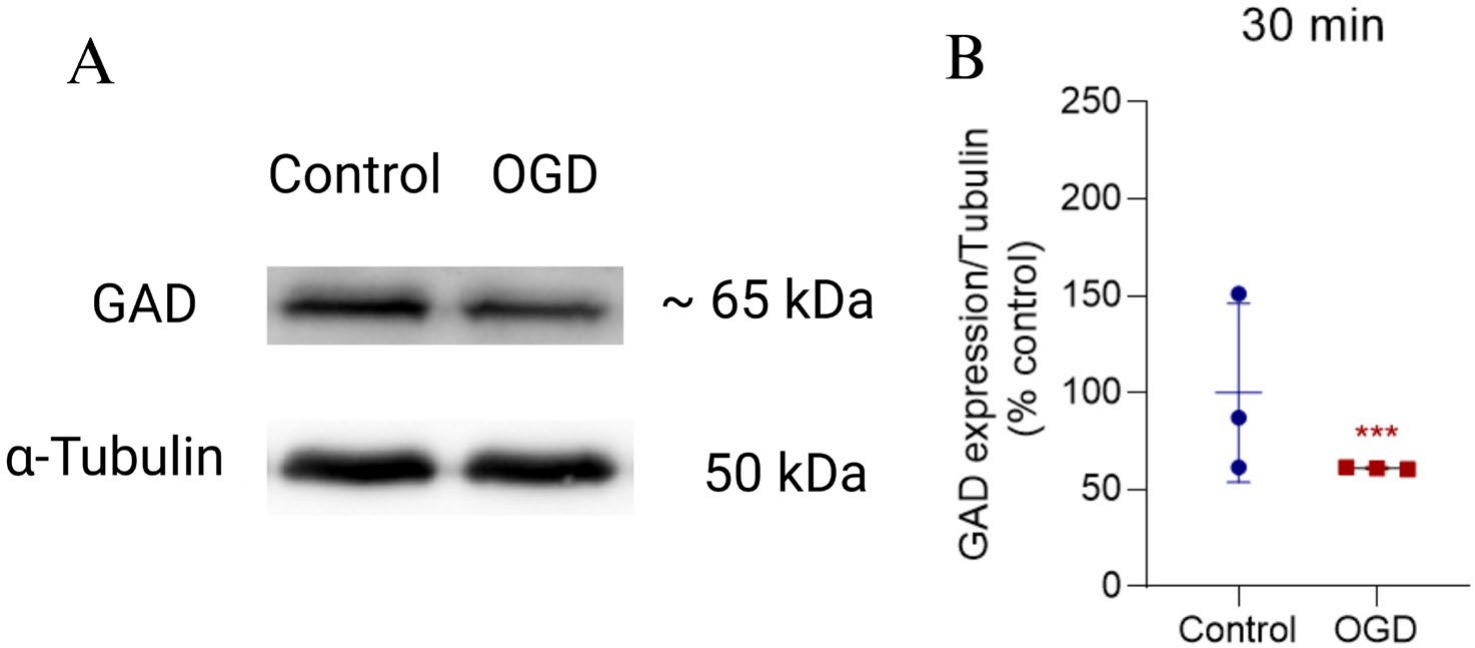
OGD reduced the levels of the rate‐limiting enzyme in GABA synthesis (GAD 65/67). (A) Representative immunoblot of GAD 65/67 expression in total protein extracts obtained from retinas subjected to OGD for 30 min. (B) Graph showing the quantification of staining intensity for GAD 65/67 normalized to α‐tubulin (% of control). A significant reduction in the expression of this enzyme was observed following OGD. Values are expressed as mean ± standard deviation (SD). Control (100.00 ± 46.19%); OGD (61.13 ± 0.59%) (*n* = 3). *t*‐test: *t*(4) = 2.927, *p* = 0.042. GAD, glutamate decarboxylase; OGD, oxygen–glucose deprivation; *p < 0.05.

We further examined the levels of GABA‐synthesizing enzyme GAD 65/67. Immunoblot analysis of the total retinal extracts revealed a significant decrease in the content of GAD in retinas exposed to OGD for 30 min (Figure 3).

The observed reduction in GAD 65/67 expression, combined with indications of decreased cell viability (Figure 1E), and reduced endogenous GABA content (Figure 2B,D2,E2) support the hypothesis of cell death among GABAergic amacrine cells. However, it cannot be ruled out that the diminished GABA expression observed in retinas exposed to OGD for at least 30 min may be from a reduced pool of synthesized GABA.

### 3.3 Involvement of GAT‐1 in OGD‐Induced Effects

**FIGURE 4:**
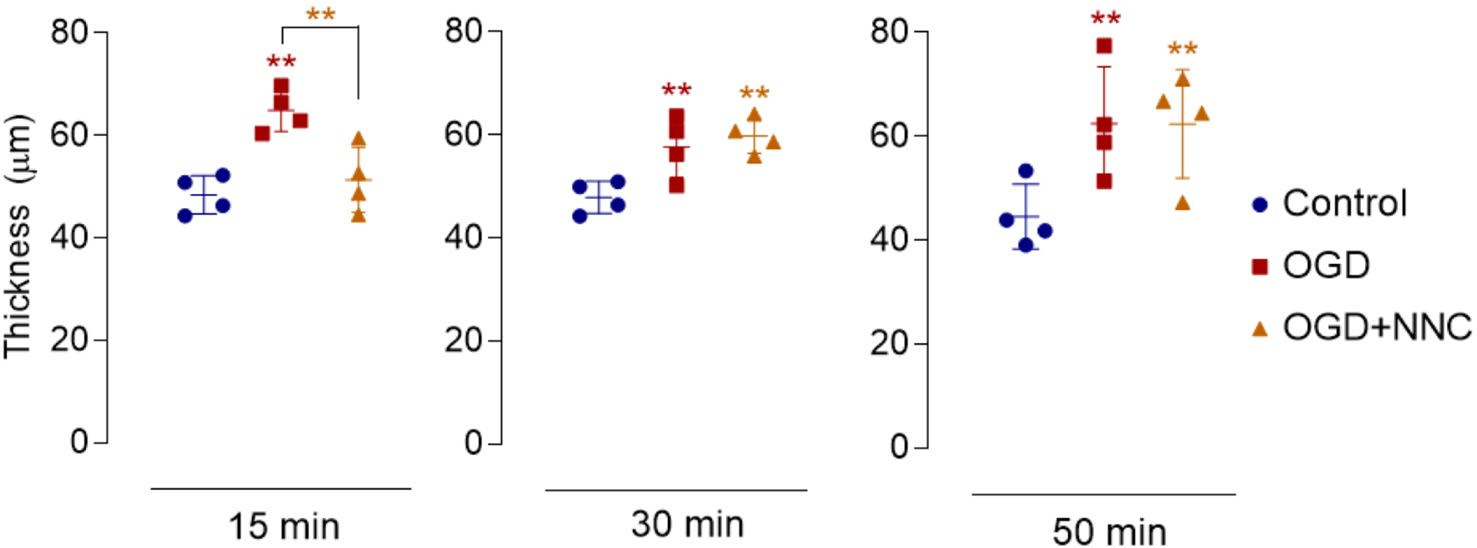
Involvement of GAT‐1 in retinal swelling. Retinal segments were subjected to OGD for 15, 30, or 50 min in the presence or absence of NNC‐711 (100 μM). The graph shows the IPL thickness (μm) measured in cross‐sections of these retinal segments. NNC‐711 partially inhibited the OGD‐induced effect at 15 min, but after 30 or 50 min of OGD, GAT‐1 did not contribute to the increase in IPL thickness. Values are expressed as mean ± standard deviation (SD). Control: 15 min (48.46 ± 3.72 μm, *n* = 4), 30 min (47.91 ± 3.10 μm, *n* = 4), 50 min (44.58 ± 6.18 μm, *n* = 4); OGD: 15 min (64.90 ± 4.06 μm, *n* = 4), 30 min (57.72 ± 5.84 μm, *n* = 4), 50 min (62.51 ± 10.94 μm, *n* = 4); OGD + NNC‐711: 15 min (51.37 ± 6.36 μm, *n* = 4), 30 min (59.87 ± 3.46 μm, *n* = 4), 50 min (62.57 ± 10.52 μm, *n* = 4). Statistical analysis using two‐way ANOVA followed by Bonferroni's multiple comparisons test showed significant differences between groups at 15 min (*p* < 0.01 compared to the control). At 15 min, a significant effect of the experimental condition was observed (*F*(2, 6) = 36.91, *p* = 0.000), along with a significant interaction with individual subjects (*F*(6, 18) = 6.001, *p* = 0.001), suggesting variability in responses across subjects. At 30 min, the experimental condition remained significant (*F*(2, 6) = 17.42, *p* = 0.003), while individual subject variability did not have a significant effect (*F*(6, 18) = 0.901, *p* = 0.516). After 50 min, the experimental condition continued to show a strong effect (*F*(2, 6) = 58.30, *p* < 0.001), with a significant interaction with sublamina (*F*(6, 18) = 15.25, *p* < 0.001). NNC‐711, GAT‐1 inhibitor; OGD, oxygen–glucose deprivation.

Next, we aimed to investigate whether the activity of GAT‐1 itself could contribute to the cellular edema induced by OGD. Given that GAT‐1 transports GABA, along with Na^+^ and Cl^−^, its function could potentially contribute to tissue swelling (Zeevalk and Nicklas 1997). To test this hypothesis, we employed the GAT‐1 inhibitor NNC‐711 (100 μM) to block GAT‐1‐mediated transport in retinas (Figure 4). Analysis of IPL thickness revealed that NNC‐711 effectively prevented the thickness increase induced by 15 min of OGD. However, after 30 or 50 min, GAT‐1 inhibition no longer prevented OGD‐induced IPL thickening. These findings suggest that GAT‐1 does not contribute to the observed swelling in retinas exposed to OGD for longer durations.

**FIGURE 5:**
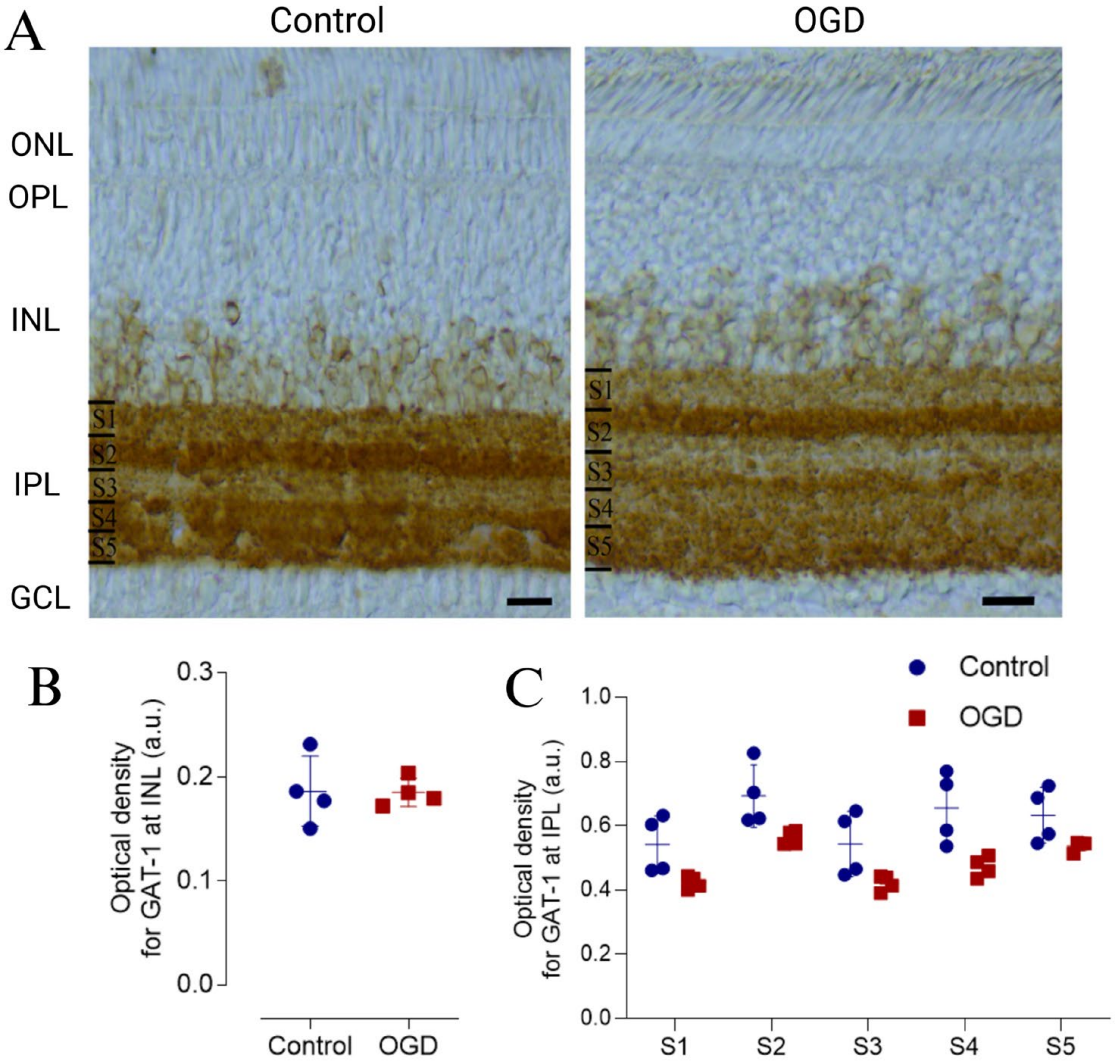
GAT‐1 expression in retinas subjected to OGD for 30 min. (A) Photomicrographs of retinal sections immunohistochemically stained for GAT‐1. Retinal segments were subjected to OGD for 30 min. (B) The graph shows the optical density (a.u.) of GAT‐1 in the INL‐resident amacrine cell population. No significant differences were observed between the control (0.186 ± 0.020 a.u., *n* = 4) and OGD (0.185 ± 0.020 a.u., *n* = 4) groups. Statistical analysis was performed using a *t*‐test (*t*(6) = 0.056, *p* = 0.956). (C) The graph shows the optical density (a.u.) of GAT‐1 in each of the five IPL sublamina (S1–S5). No subgroup‐specific differences were detected. Values are expressed as mean ± standard deviation (SD). Control: S1 (0.54 ± 0.08), S2 (0.69 ± 0.10), S3 (0.54 ± 0.10), S4 (0.65 ± 0.10), S5 (0.63 ± 0.08); OGD: S1 (0.42 ± 0.02), S2 (0.56 ± 0.02), S3 (0.42 ± 0.02), S4 (0.47 ± 0.02), S5 (0.53 ± 0.02) (*n* = 4 for all groups). Statistical analysis was performed using a two‐way ANOVA followed by Bonferroni's multiple comparisons test. The two‐way ANOVA showed significant effects of sublamina (*F*(4, 30) = 6.598, *p* = 0.001) and treatment (*F*(1, 30) = 33.42, *p* < 0.001), but no significant interaction between factors (*F*(4, 30) = 0.418, *p* = 0.794). a.u., arbitrary units; GAT‐1, type 1 GABA transporter; OGD, oxygen–glucose deprivation; Sn, sublamina. Scale bar = 20 μm.

Since GAT‐1 did not appear to contribute to retinal swelling after 30 min of OGD, we investigated whether the lack of involvement could be attributed to changes in the expression pattern of this transporter. To examine this, we performed an optical density analysis of GAT‐1 in each of the five sublaminae of IPL as well as INL (Figure 5). Our analysis revealed that OGD did not alter GAT‐1 labeling intensity in the INL (Figure 5B). However, when assessing the intensity of GAT‐1 immunostaining in the IPL, we observed that the treatment (control vs. OGD) contributed significantly to the variance in GAT‐1 immunostaining in retinas exposed to OGD for 30 min (Figure 5C, Table 2, *p* = 0.0007). Furthermore, our analysis demonstrated that the observed variance was influenced by the specific sublamina within the IPL (Table 2, *p* = 0.0043), although no significant differences were detected between the control and OGD groups when comparing each sublamina individually (Table 2).

**TABLE 2:** Labeling intensity values for GAT‐1 in IPL.

Lamina	Control ± SD (a.u.)	OGD ± SD (a.u.)	*n*
S1	0.562 ± 0.084	0.429 ± 0.026	4
S2	0.696 ± 0.014	0.556 ± 0.026	
S3	0.577 ± 0.136	0.428 ± 0.028	
S4	0.680 ± 0.096	0.483 ± 0.046	
S5	0.651 ± 0.072	0.529 ± 0.030	

Abbreviations: a.u., arbitraryunit; GAT‐1, GABA transporter type 1; IPL, inner plexiform layer; OGD, glucose and oxygen deprivation; SD, standard derivation; Sn, sublamina n.

### 3.4 Picrotoxin Exerts a Neuroprotective Effect on Retinas Subjected to OGD

**FIGURE 6:**
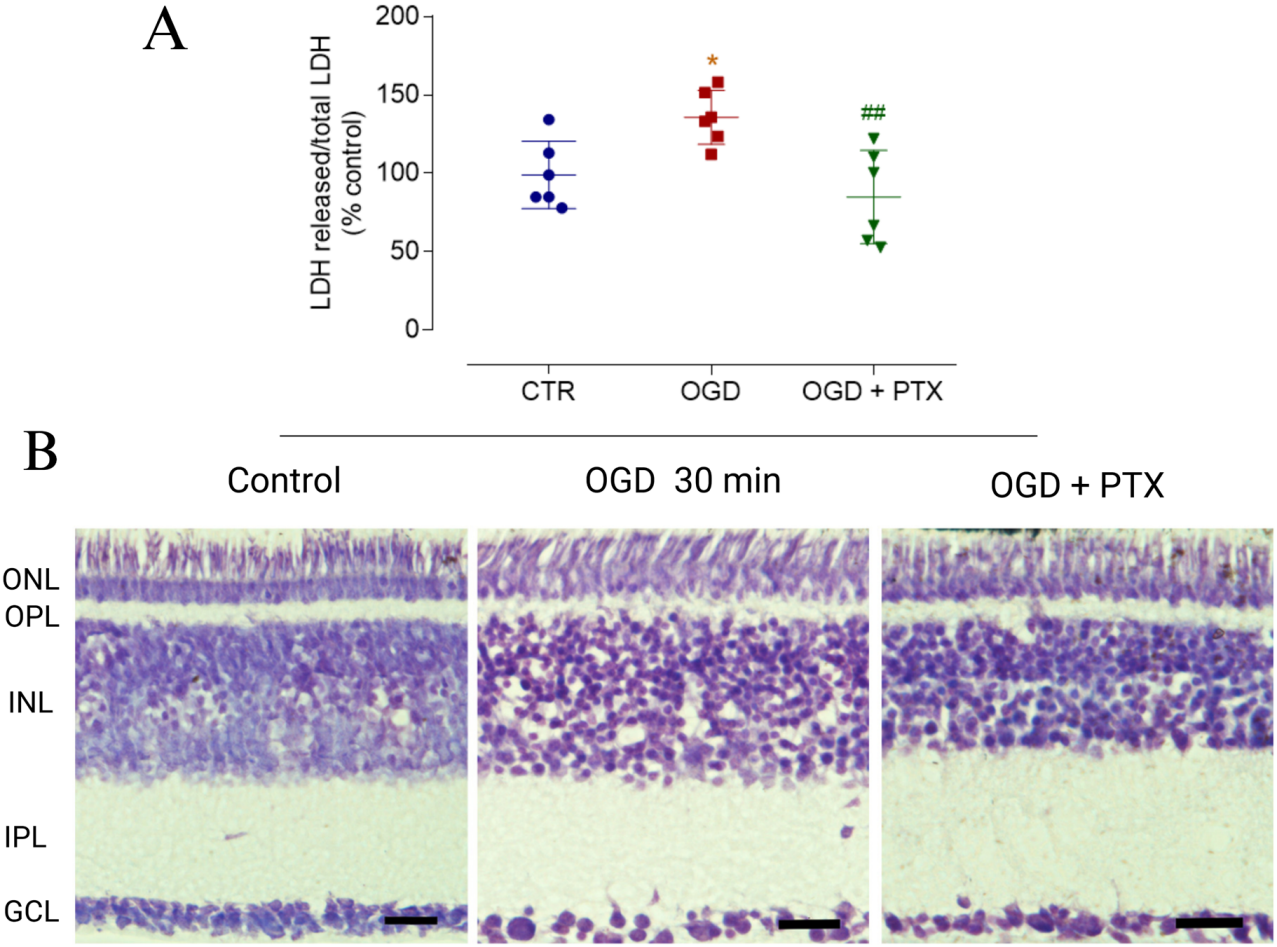
Protective effect of PTX on retinas subjected to OGD. (A) Cell death was assessed by measuring LDH activity in the extracellular medium, expressed as the ratio of released LDH to total LDH, in retinal segments treated or not with PTX (200 μM) and subjected to OGD for 30 min. The graph shows that PTX treatment completely prevented the OGD‐induced increase in LDH release relative to the control. Values are expressed as mean ± standard deviation (SD): Control = 100.00 ± 21.43% (*n* = 6), OGD = 135.60 ± 17.10% (*n* = 6), OGD + PTX = 84.93 ± 29.74% (*n* = 6). Statistical analysis was performed using a two‐way ANOVA followed by Bonferroni's multiple comparisons test. The analysis revealed a significant effect of the independent factor (*F*(2, 10) = 14.25, *p* = 0.001), indicating that the treatment groups differed significantly. However, the grouping factor did not significantly affect the results (*F*(5, 10) = 2.097, *p* = 0.149). (B) Photomicrographs of retinal sections subjected to Nissl staining. Retinal segments were exposed to the same experimental conditions as described above. Qualitative analysis revealed that PTX attenuated disorganization and vacuole formation in the INL but did not prevent swelling in the IPL. LDH, lactate dehydrogenase; OGD, oxygen–glucose deprivation; PTX, picrotoxin. Significance: *p < 0.05 compared to the control; ^##^ *p* < 0.01 compared to OGD. Scale bar: 20 μm.

Considering that GABA can be released during OGD, either due to cell death or stimulated release via GAT‐1, we then aimed to investigate the potential role of GABA released during OGD. To this end, 200 μM picrotoxin (PTX), a nonselective antagonist of GABAergic ionotropic receptors, was applied to evaluate the extent of cell death induced by OGD related to these receptors. The cell viability assay revealed that PTX significantly mitigated the cell death induced by 30 min of OGD (Figure 6A). A control experiment using PTX alone was performed, showing no significant effect on cell viability (PTX: 115.50 ± 9.97%, *n* = 3). Morphological analysis of PTX‐treated retinas exposed to OGD showed a more organized INL. Notably, the qualitative analysis suggests that PTX treatment did not prevent OGD‐induced IPL swelling (Figure 6B).

**FIGURE 7:**
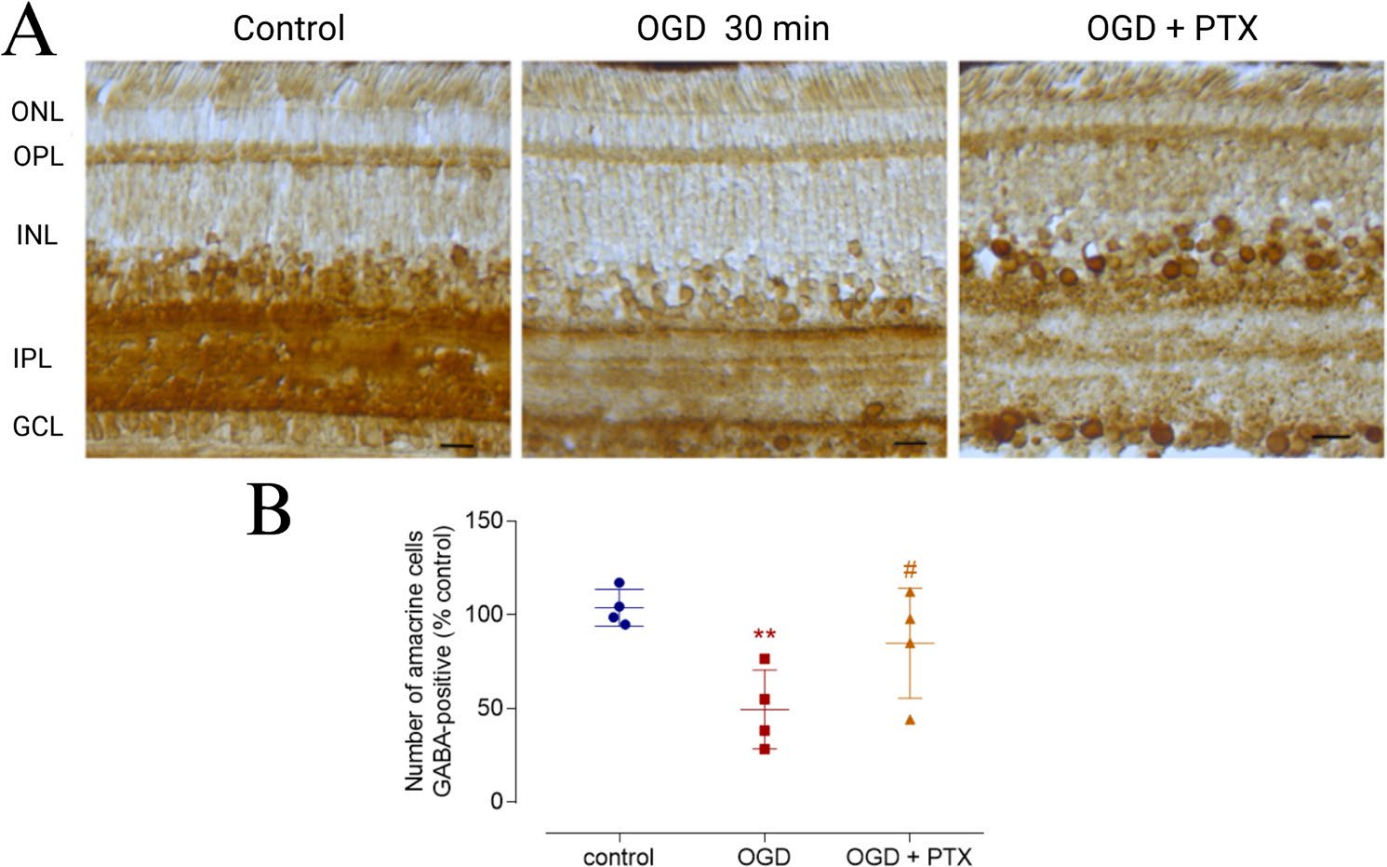
Effect of PTX on GABAergic amacrine cell populations in retinas subjected to OGD. (A, B) Retinal segments were treated or not with PTX (200 μM) and subjected to OGD for 30 min. (A) Photomicrographs of retinas immunostained for GABA. PTX treatment partially prevented the OGD‐induced reduction in GABA immunostaining. (B) The graph shows the number of GABA‐positive amacrine cells in different experimental groups. A significant reduction in the number of GABA‐positive amacrine cells was observed in retinas subjected to OGD, which was partially mitigated by PTX treatment, suggesting a neuroprotective role for PTX. Values are expressed as mean ± standard deviation (SD): Control = 100.00 ± 9.76%, OGD = 49.56 ± 21.06% (*n* = 4), OGD + PTX = 84.96 ± 29.36% (*n* = 4). The average number of amacrine cells counted per field under control conditions was 152.35 cells. Statistical analysis was performed using a two‐way ANOVA followed by Bonferroni's multiple comparisons test. The two‐way ANOVA revealed significant effects of the interaction between factors (*F*(6, 18) = 57.94, *p* < 0.001), time (*F*(2.010, 12.06) = 13.59, *p* = 0.001), and treatment condition (*F*(2, 6) = 728.6, *p* < 0.001). However, individual subject variability did not significantly affect the results (*F*(6, 18) = 0.365, *p* = 0.892). OGD, oxygen–glucose deprivation; PTX, picrotoxin. Significance: **p < 0.01 compared with the control. Scale bar: 20 μm.

Given the neuroprotective effects of PTX in retinas subjected to 30 min of OGD, we aimed to evaluate whether GABAergic amacrine cells may benefit from this protective action since we found a reduction in this population following OGD. Our analysis revealed that PTX treatment partially counteracted the OGD‐induced decrease in the number of GABAergic amacrine cells (Figure 7A,B).

### 3.5 Bumetanide Promoted Neuroprotection in Retinas Subjected to OGD

**FIGURE 8:**
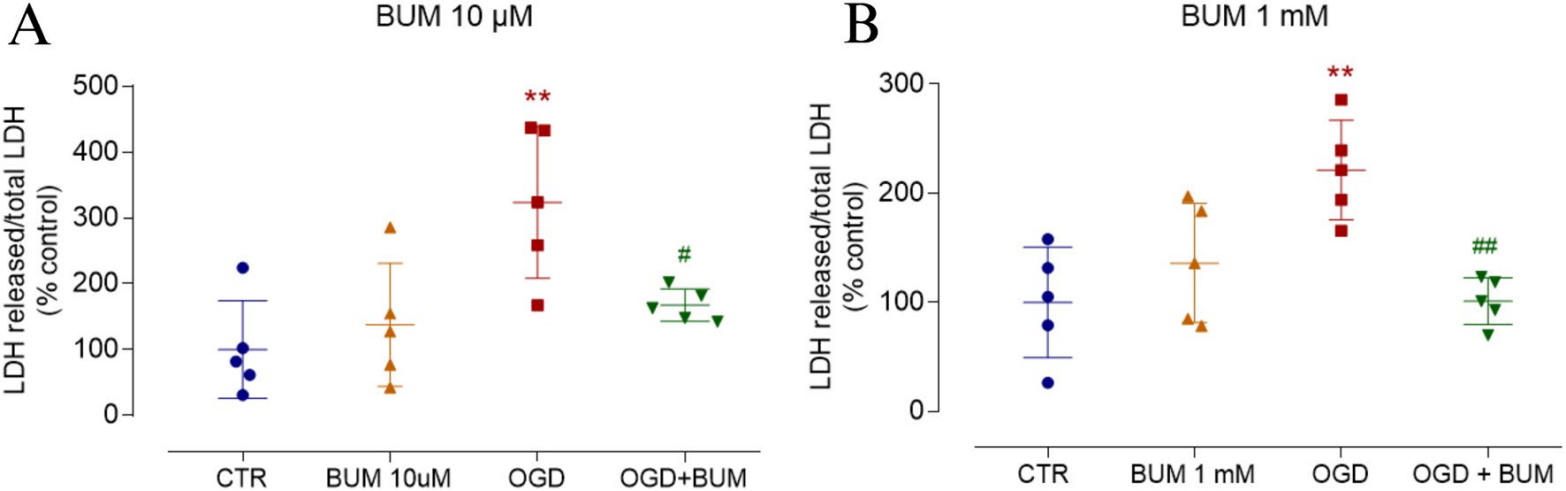
Bumetanide promotes neuroprotection in retinas subjected to OGD. (A, B) Retinas were treated with BUM at a concentration that selectively inhibits the NKCC1 transporter (10 μM, A) or at a higher concentration that blocks both NKCC1 and KCC2 transporters (1 mM, B). Cell death was assessed by measuring LDH activity in the extracellular medium (released LDH/total LDH). (A) The graph shows that treatment with 10 μM BUM prevented the OGD‐induced increase in LDH release. Values are expressed as mean ± standard deviation (SD). Control: 100.00 ± 74.43% (*n* = 5), BUM (10 μM): 137.60 ± 93.67% (*n* = 5), OGD: 324.10 ± 133.52% (*n* = 5), OGD + BUM (10 μM): 167.30 ± 24.61% (*n* = 5). Statistical analysis was performed using a two‐way ANOVA followed by Bonferroni's multiple comparisons test. The two‐way ANOVA revealed a significant main effect of the independent factor (*F*(3, 12) = 13.04, *p* < 0.001), indicating significant differences between treatment groups. However, the grouping factor did not significantly affect the results (*F*(4, 12) = 3.124, *p* = 0.056). (B) The graph shows that treatment with 1 mM BUM blocked the OGD‐induced increase in LDH release. Values are expressed as mean ± SD: Control: 100.00 ± 50.59% (*n* = 5), BUM (1 mM): 136.10 ± 70.54% (*n* = 5), OGD: 221.20 ± 58.83% (*n* = 5), OGD + BUM (1 mM): 101.10 ± 27.69% (*n* = 5). Statistical analysis was performed using a two‐way ANOVA, which revealed a significant main effect of the independent factor (*F*(3, 12) = 9.349, *p* = 0.002), indicating significant differences between treatment groups. However, the grouping factor did not significantly affect the results (*F*(4, 12) = 0.188, *p* = 0.940). Significance: *p < 0.05 compared with the control; ^##^ *p* < 0.05 compared to OGD. BUM, bumetanide; KCC2, K^+^‐Cl^−^ cotransporter 2; LDH, lactate dehydrogenase; NKCC1, Na^+^‐K^+^‐Cl^−^ cotransporter 1; OGD, oxygen–glucose deprivation.

Evidence suggests that ischemic conditions in CNS may alter the expression patterns of NKCC1 or KCC2, potentially contributing to excitotoxic effects (Nascimento et al. 2024). Based on this, our objective was to investigate the involvement of NKCC1 and KCC2 in OGD‐induced cell death. To achieve this, retinas subjected to OGD were treated simultaneously with either 10 μM bumetanide (BUM) to selectively inhibit the NKCC1 transporter (Figure 8A) or 1 mM BUM (Figure 8B), a nonselective concentration that inhibits both NKCC1 and KCC2. These concentrations were chosen based on previous studies (Payne et al. 2003) to distinguish the effect of KCC2 inhibition from that of selective NKCC1 blockade. Interestingly, both BUM concentrations exhibited similar inhibitory effects on cell death induced by 30 min of OGD (Figure 8). These findings highlight the involvement of chloride flux, in which the direction is established by the electrochemical gradient set by chloride cotransporters, in the pathophysiology of ischemia.

**FIGURE 9:**
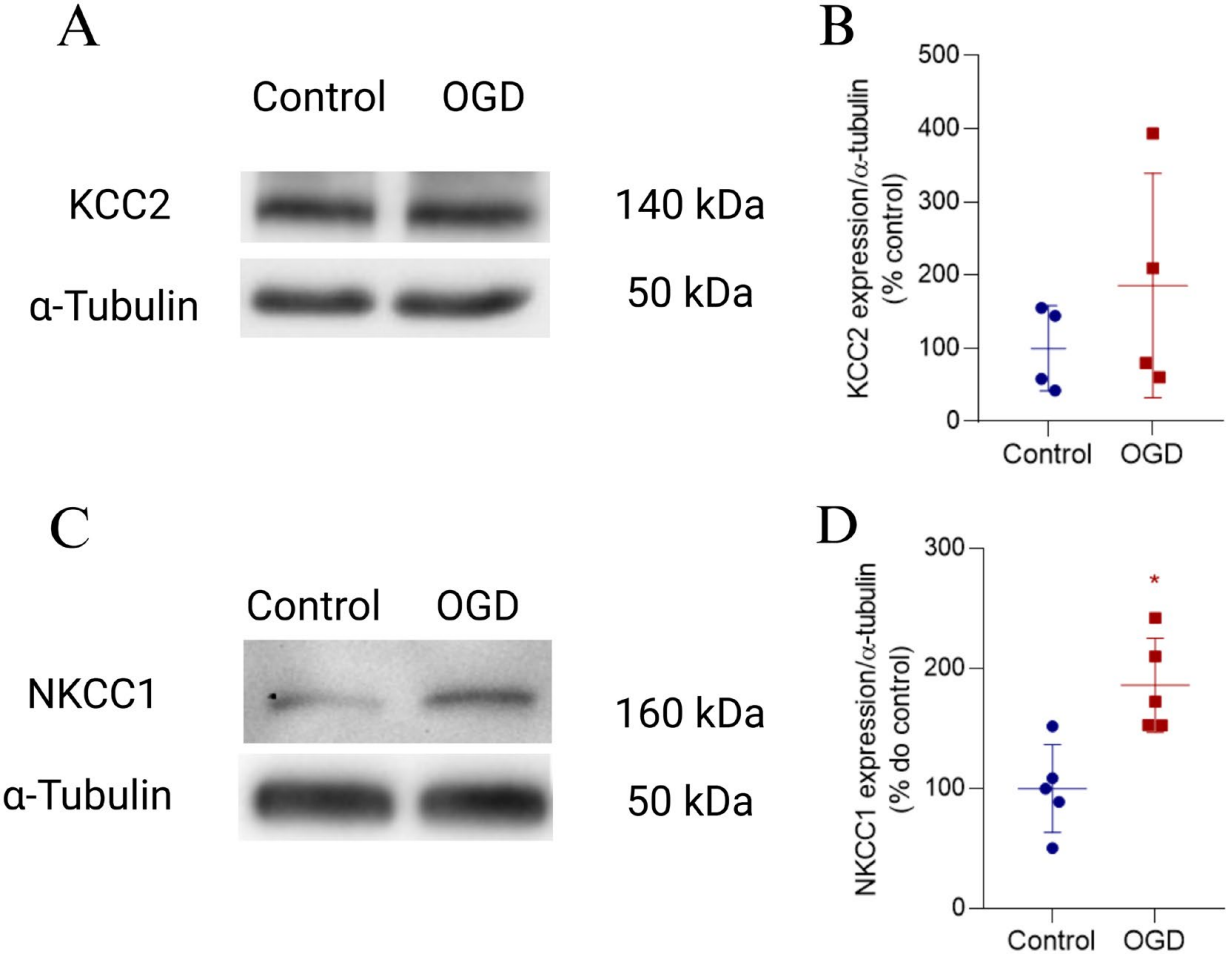
OGD did not alter KCC2, but increased NKCC1 protein levels. (A, C) Representative immunoblots of KCC2 and NKCC1, respectively, in total protein extracts from retinas subjected to 30 min of OGD. (B) Graph showing the quantification of staining intensity for KCC2 normalized to α‐tubulin (% of control), indicating no significant difference in KCC2 content between the control and OGD groups. Values are expressed as mean ± standard deviation (SD): Control: 100.00 ± 58.00%; OGD: 185.70 ± 153.46% (*n* = 4). Statistical analysis using an unpaired *t*‐test showed no significant difference between groups (*t*(6) = 1.166, *p* = 0.290). (D) Graph showing the quantification of NKCC1/α‐tubulin staining intensity (% of control), indicating an increase in NKCC1 levels in retinas subjected to OGD. Control: 100.00 ± 42.20%; OGD: 178.40 ± 33.60% (*n* = 4). Statistical analysis using an unpaired *t*‐test confirmed a significant difference between groups (*t*(6) = 3.795, *p* = 0.009). KCC2, K^+^‐Cl^−^ cotransporter type 2; NKCC1, Na^+^‐K^+^‐Cl^−^ cotransporter type 1; OGD, oxygen–glucose deprivation; *p < 0.05.

To elucidate the mechanism underlying the protective effect of BUM on cell death, we evaluated the patterns of NKCC1 and KCC2 levels in the retinas exposed to 30 min of OGD. Immunoblot analysis of total retinal extracts revealed no significant difference in KCC2 protein content between control and OGD‐treated retinas (Figure 9A,B). In contrast, NKCC1 protein levels significantly increased, approximately doubling, in retinas subjected to OGD (Figure 9C,D). Therefore, the data indicate that OGD alters the balance of chloride transport and, possibly, its electrochemical gradient, producing an excitatory response of GABA related to cell death promoted by this pathological condition.

## 4 Discussion

The present study uses an ex vivo model that preserves retinal architecture, allowing us to capture early and progressive changes induced by OGD, a model applied to evaluate the early effects of retinal ischemia. The findings with 15 min OGD indicate that in early stages of ischemia occur IPL thickness increases, which is considered a sign of retinal swelling, and GABA release via GAT‐1, with no retinal cell death. The rapid onset of OGD‐induced swelling aligns with the suggested role of ion transport mechanisms in the early phases of ischemic damage (Romano et al. 1998; Zeevalk and Nicklas 1990). In addition, it was shown that swelling caused by metabolic stress is attenuated by noncompetitive GAT‐1 inhibitors (Zeevalk and Nicklas 1997). In the present study, NNC‐711, a noncompetitive inhibitor of GAT‐1, prevented IPL edema, strengthening the idea of a possible contribution of the transporter in retinal swelling. The blockage of GAT‐1 failed to protect against swelling in 30 or 50 min OGD, indicating that other mechanisms, such as glutamate excitotoxicity, may dominate with prolonged ischemic stress (Choi and Rothman 1990; Kostandy 2012).

The longer the OGD period, the worse the damage. OGD for 30 min promotes visible signs of tissue injury, including swelling, disorganization of nuclear layers, a significant decrease in cell viability, and an intense reduction in GABA content in INL and IPL. The same parameters were even more pronounced in retinas subjected to OGD for 50 min. It is known that ischemia leads to excitotoxic damage through glutamatergic signaling (Choi and Rothman 1990; Kostandy 2012; Lai et al. 2014; Shen et al. 2022). Zeevalk and Nicklas's 1990s studies demonstrated that different ischemic models in chick embryo retinas for 30–45 min resulted in histopathological changes and release of GABA mediated by the NMDA receptor activation (Zeevalk and Nicklas 1990, 1991, 1997). Previous research by our group shows that GABAergic retinal cells exposed to excitatory amino acids, glutamate and its ionotropic receptors agonists, or low glucose for 30 min release their GABA content via GAT‐1 reversion, without cell death (Calaza et al. 2001; Miya‐Coreixas et al. 2013). Therefore, the release of GABA induced by OGD is probably related to the increase in extracellular glutamate availability during ischemia. We found that PTX exerts a neuroprotective effect on retinas subjected to OGD, as evidenced by its ability to inhibit retinal swelling and prevent the reduction in cell viability. PTX's mitigates cellular damage and swelling likely by decreasing chloride ion (Cl^−^) flux through ionotropic GABA receptors. These findings reinforce previous observations that GABAergic signaling contributes to ionic imbalance under ischemic conditions (Chen et al. 1998, 1999; Wässle et al. 2009). Accordingly, Bumetanide (BUM), a NKCC1 inhibitor, also demonstrated neuroprotective properties in retinas exposed to OGD, consistent with studies highlighting NKCC1's involvement in ischemic excitotoxicity (Beck et al. 2003; Hama et al. 2008; Schulte et al. 2018; Ben‐Ari and Cherubini 2022; Nascimento et al. 2024). OGD‐induced upregulation of NKCC1 levels, observed here and in other studies, changes the electrochemical gradient for chloride, which favors an efflux of Cl^−^ when ionotropic GABA receptors are activated, leading to an increase in the excitatory response. In addition, no alteration was detected in retinal KCC2 content, strengthening the idea that NKCC1 plays a dominant role in disrupting ionic homeostasis in this context. Hence, this change in chloride cotransporters promoted by OGD, together with GABA release, builds up the glutamate‐induced excitotoxicity and cell death during ischemia (Schomberg et al. 2001; Chen and Sun 2005). Therefore, BUM's neuroprotective effects are likely due to its inhibition of NKCC1‐mediated Cl^−^ influx, preventing the intracellular Cl^−^ accumulation and subsequent neuronal depolarization intensification by chloride‐permeable ionotropic receptors (Pond et al. 2006; Jaggi et al. 2015).

Interestingly, GABAergic displaced amacrine cells show no alteration in the number of GABA‐positive cells after any time of OGD, suggesting a greater resistance of this cell population to this insult. It is worth noting that this cell population does not present GAT‐1 (Calaza Kda et al. 2003), suggesting a different mechanism of GABA release that could contribute to a distinct response in ischemic conditions, probably related to ion transport, as suggested before (Zeevalk and Nicklas 1997; Hama et al. 2008). Even though the explanation for this feature is unclear, the possibility that less GABA is released in the ganglion cell layer, together with the hypothesis that GABA is intensifying excitotoxicity during ischemic events and that it has been shown that NKCC1 content is increased in rat retina ganglion cells after ischemia (Kim and Ahn 2012), suggests that cells in the ganglion cell layer could be more resistant to ischemia.

Concerning the reduction in GAD 65/67 levels detected after OGD, further investigation is necessary to determine whether surviving GABAergic cells in the postinjury retina exhibit changes in the expression or activity of GAD 65/67, to evaluate the impact of the loss of a subset of the GABAergic population. In addition, previous research has shown that treatment with glutamate, kainate, or GABA for 16 h decreases GAD 65/67 levels in chick retinas, while 1 h is sufficient to reduce GAD activity (de Almeida et al. 2002). Thus, the reduction in GAD 65/67 content in retinas exposed to OGD for 30 min could arise from glutamate or even GABA signaling, suggesting that a similar modulation could occur in ischemic conditions and/or in shorter exposures. However, a more detailed analysis must be performed to clarify the mechanism(s) involved.

Interestingly, PTX's neuroprotective effects may also involve glycine receptors, as glycine release during OGD (Harsing et al. 2012). The presence of dual GABA/glycine inhibitory signaling in amacrine cells suggests a complex interplay during ischemia, where PTX may inhibit glycinergic as well as GABAergic pathways (Pérez‐León et al. 2022). This aligns with evidence indicating that Cl^−^ influx via glycine receptors contributes to retinal swelling and excitotoxicity (Lynch 2004; Yang et al. 2007; Breitinger and Breitinger 2020).

## 5 Concluding Remarks

In conclusion, our results provide evidence that early interventions targeting ion transport mechanisms, such as NKCC1 and GABAergic pathways, can mitigate retinal damage caused by ischemic stress. PTX and BUM, by modulating these pathways, offer promising therapeutic potential for ischemic retinopathies. The interplay between excitotoxicity, ionic dysregulation, and neurotransmitter signaling offers a framework for future studies aiming to unravel the complex dynamics of neuronal injury and repair. These findings contribute to the understanding of ischemic retinal injury and highlight novel targets for preserving vision in conditions such as retinal ischemia and stroke.

A limitation of the present study includes the lack of in vivo validation. Future studies should confirm these findings in vivo and further investigate the interplay between NKCC1 and GAT‐1 in ischemic retinal damage.

## Author Contributions

K.C.C. had the original idea, designed the experiments, was responsible for the funding acquisition, administered the whole project, reviewed the manuscript, and supervised. A.A.N. and V.S.M.‐C. contributed to the methodology, data collection, data analysis, wrote the original draft, and reviewed the manuscript. T.H.O.N., D.S.M.A., and G.F.S. were involved in data collection, data analysis, and reviewing and editing the manuscript. R.B. contributed to the methodology, funding acquisition, and review and editing of the manuscript. All authors performed a literature search, read, and agreed to the published version of the manuscript.

## Ethics Statement

The authors have nothing to report.

## Consent

The authors have nothing to report.

## Conflicts of Interest

The authors declare no conflicts of interest.

## Declaration of Transparency

The authors, reviewers and editors affirm that in accordance to the policies set by the *Journal of Neuroscience Research*, this manuscript presents an accurate and transparent account of the study being reported and that all critical details describing the methods and results are present.

## Supporting information


Table S1.

**Data S1.** Transparent Science Questionnaire for Authors

## Data Availability

Data available on request from the authors. The data that support the findings of this study are available from the corresponding author upon reasonable request.
