# Supplementary material for: Modulation of GABAergic System in a Chicken Retinal Ischemic Model: The Role of Chloride Cotransporters
Source: J Neurosci Res. 2025 May 12;103(5):e70043. doi: 10.1002/jnr.70043 (PMC12067523; doi:10.1002/jnr.70043)
Supplement: Supplementary file 2 [file JNR-103-e70043-s001.docx]

Antibody	Immunogen Structure	Manufacturer, Catalog #, RRID, Species, Type	Concentration used
anti-GABA	γ-aminobutyric acid (GABA) conjugated to BSA	Sigma-Aldrich (USA), Cat# A2052, RRID: AB_477652, Rabbit, Polyclonal primary antibody	1:7000
anti-GAT-1	Rat GAT-1, C-terminus (aa 588-599)	Millipore/Sigma-Aldrich (USA), Cat# AB1570W, RRID: AB_90791, rabbit, polyclonal primary antibody	1:100
Anti-goat	Recognizes both heavy and light chains (H+L)	Vector Laboratories (USA), Cat# BA-5000, RRID:AB_2336126, goat, polyclonal biotinylated secondary antibody	1:200
anti-KCC2	N-terminal His-tag fusion protein corresponding to residues 932-1043 of rat K+/Cl- Cotransporter (KCC2).	Millipore/Sigma-Aldrich (USA), Cat# 07-432, RRID: AB_310611, Rabbit, polyclonal primary antibody	1:500
anti-NKCC1	Recombinant 38kDa peptide fragment of carboxy terminus encoded by cDNA from T84 (human) epithelial Na-K-Cl cotransport protein	DSHB (USA), Cat# t4, RRID: AB_528406, Mouse, monoclonal primary antibody	1:1000
Anti-rabbit	Recognizes both heavy and light chains (H+L)	Vector Laboratories (USA), Cat# BA-1000, RRID: AB_2313606, rabbit, polyclonal biotinylated secondary antibody	1:200
Anti-Tubulin	Recognizes an epitope located at the C-terminal end of the α-tubulin isoform in various organisms	Sigma-Aldrich (USA), Cat# T5168, RRID: AB_477579, mouse, monoclonal primary antibody	1:70000
Anti-rabbit	IgG; Recognizes both heavy and light chains (H+L)	Amersham/Cytiva (United Kingdom), Cat# NA934, RRID: AB_772206, rabbit, polyclonal-HRP,	1:10.000
Anti-mouse	IgG; Recognizes both heavy and light chains (H+L)	Amersham/Cytiva (United Kingdom), Cat# NA931, RRID: AB_772210, mouse, polyclonal HRP secondary antibody	1: 10.000
Anti-sheep	IgG; Recognizes both heavy and light chains (H+L)	Invitrogen/Thermo Fisher Scientific (USA), Cat# 61-8620, RRID: AB_2533942, sheep, polyclonal HRP secondary antibody	1:1000
